# Systematic ocular phenotyping of 8,707 knockout mouse lines identifies genes associated with abnormal corneal phenotypes

**DOI:** 10.1186/s12864-025-11222-8

**Published:** 2025-01-20

**Authors:** Peter Vo, Denise M. Imai-Leonard, Benjamin Yang, Andrew Briere, Andy Shao, M. Isabel Casanova, David Adams, Takanori Amano, Oana Amarie, Zorana Berberovic, Lynette Bower, Robert Braun, Steve Brown, Samantha Burrill, Soo Young Cho, Sharon Clementson-Mobbs, Abigail D’Souza, Mary Dickinson, Mohammad Eskandarian, Ann M. Flenniken, Helmut Fuchs, Valerie Gailus-Durner, Jason Heaney, Yann Hérault, Martin Hrabe de Angelis, Chih-Wei Hsu, Shundan Jin, Russell Joynson, Yeon Kyung Kang, Haerim Kim, Hiroshi Masuya, Hamid Meziane, Steve Murray, Ki-Hoan Nam, Hyuna Noh, Lauryl M. J. Nutter, Marcela Palkova, Jan Prochazka, Miles Joseph Raishbrook, Fabrice Riet, Jennifer Ryan, Jason Salazar, Zachery Seavey, John Richard Seavitt, Radislav Sedlacek, Mohammed Selloum, Kyoung Yul Seo, Je Kyung Seong, Hae-Sol Shin, Toshihiko Shiroishi, Michelle Stewart, Karen Svenson, Masaru Tamura, Heather Tolentino, Uchechukwu Udensi, Sara Wells, Jacqueline White, Amelia Willett, Janine Wotton, Wolfgang Wurst, Atsushi Yoshiki, The International Mouse Phenotyping Consortium, Louise Lanoue, K. C. Kent Lloyd, Brian C. Leonard, Michel J. Roux, Colin McKerlie, Ala Moshiri

**Affiliations:** 1 California Northstate University College of Medicine, Elk Grove, CA USA; 2 Department of Pathology, Microbiology & Immunology, School of Veterinary Medicine, University of California Davis, Sacramento, CA USA; 3 University of California Davis School of Medicine, Sacramento, CA USA; 4 Touro University California College of Medicine, Vallejo, CA USA; 5 Department of Ophthalmology & Vision Science, School of Medicine, University of California Davis, Sacramento, CA USA; 6 Department of Surgical and Radiological Sciences, School of Veterinary Medicine, University of California Davis, Davis, CA USA; 7 The Wellcome Trust Sanger Institute, Wellcome Genome Campus, Hinxton, Cambridge, UK; 8 RIKEN BioResource Research Center, Tsukuba, Japan; 9 Institute of Experimental Genetics, German Mouse Clinic, Helmholtz Zentrum München, Neuherberg, Germany; 10 The Centre for Phenogenomics, Lunenfeld-Tanenbaum Research Institute, Mount Sinai Hospital, Toronto, ON Canada; 11 Mouse Biology Program, University of California Davis, Davis, CA USA; 12 The Jackson Laboratory, Bar Harbor, ME USA; 13 Medical Research Council, Harwell Institute, Harwell, UK; 14 Department of Molecular and Life Science, Hanyang University, Seoul, Republic of Korea; 15 Mary Lyon Centre, Medical Research Council, Harwell Institute, Harwell, UK; 16 Department of Integrative Physiology, Baylor College of Medicine, Houston, TX USA; 17 Department of Molecular and Human Genetics, Baylor College of Medicine, Houston, TX USA; 18 Université de Strasbourg, CNRS UMR 7104, INSERM U 1258, IGBMC, Institut Clinique de la Souris, PHENOMIN, Illkirch-Graffenstaden, France; 19 College of Veterinary Medicine, Seoul National University, Seoul, Republic of Korea; 20 Laboratory Animal Center, Korea Research Institute of Bioscience and Biotechnology, Daejeon, Republic of Korea; 21 The Centre for Phenogenomics, The Hospital for Sick Children, Toronto, ON Canada; 22 Czech Centre for Phenogenomics, Institute of Molecular Genetics of the Czech Academy of Sciences, Vestec, Czech Republic; 23 Department of Ophthalmology, Institute of Vision Research, Yonsei University College of Medicine, Seoul, Republic of Korea; 24 Laboratory of Developmental Biology and Genomics, Research Institute of Veterinary Science, BK21 Plus Program for Advanced Veterinary Science, College of Veterinary Medicine and Interdisciplinary Program for Bioinformatics, Seoul National University, Seoul, Republic of Korea; 25 Institute of Developmental Genetics, Helmholtz Zentrum München, Neuherberg, Germany; 26 Department of Surgery, School of Medicine, University of California Davis, Sacramento, CA USA; 27 Université de Strasbourg, CNRS, Inserm, IGBMC UMR 7104- UMR-S 1258, Illkirch, F-67400 France; 28 Department of Laboratory Medicine & Pathobiology, Faculty of Medicine, University of Toronto, Toronto, ON Canada; 29 UC Davis Eye Center, 4860 Y St., Ste, Sacramento, CA 2400, 95817 USA

**Keywords:** Corneal dysmorphologies, Corneal disease, Corneal dystrophies

## Abstract

**Purpose:**

Corneal dysmorphologies (CDs) are typically classified as either regressive degenerative corneal dystrophies (CDtrs) or defective growth and differentiation-driven corneal dysplasias (CDyps). Both eye disorders have multifactorial etiologies. While previous work has elucidated many aspects of CDs, such as presenting symptoms, epidemiology, and pathophysiology, the genetic mechanisms remain incompletely understood. The purpose of this study was to analyze phenotype data from 8,707 knockout mouse lines to identify new genes associated with the development of CDs in humans.

**Methods:**

8,707 knockout mouse lines phenotyped by the International Mouse Phenotyping Consortium were queried for genes associated with statistically significant (*P* < 0.0001) abnormal cornea morphology to identify candidate CD genes. Corneal abnormalities were investigated by histopathology. A literature search was used to determine the proportion of candidate genes previously associated with CDs in mice and humans. Phenotypes of human orthologues of mouse candidate genes were compared with known human CD genes to identify protein-protein interactions and molecular pathways using the Search Tool for the Retrieval of Interacting Genes/Proteins (STRING), Protein Analysis Through Evolutionary Relationships (PANTHER), and Kyoto Encyclopedia of Genes and Genomes.

**Results:**

Analysis of data from 8,707 knockout mouse lines identified 213 candidate CD genes. Of these, 37 (17%) genes were previously known to be associated with CD, including 14 in the mouse, 16 in humans, and 7 in both. The remaining 176 (83%) genes have not been previously implicated in CD. We also searched publicly available RNAseq data and found that 131 of the total 213 (61.5%) were expressed in adult human corneal tissue. STRING analysis showed several interactions within and between candidate and established CD proteins. All cellular pathways of the established genes were found in the PANTHER analysis of the candidate genes. Several of the candidate genes were implicated in corneal disease, such as TGF-ß signaling. We also identified other possible underappreciated mechanisms relevant to the human cornea.

**Conclusions:**

We identified 213 mouse genes that resulted in statistically significant abnormal corneal phenotypes in knockout mice, many of which have not previously been implicated in corneal pathology. Bioinformatic analyses implicated candidate genes in several signaling pathways which are potential therapeutic targets.

**Supplementary Information:**

The online version contains supplementary material available at 10.1186/s12864-025-11222-8.

## Introduction

Corneal dysmorphologies (CDs) are a group of acquired but predominantly genetically inherited eye disorders that cause progressive vision loss and can be associated with systemic abnormalities [1]. Patients can be asymptomatic or can present with complaints of blurry vision, eye pain, light sensitivity, and foreign body sensation [2]. Given the variation in clinical presentation it is thought that both categories of CDs (corneal dystrophies; CDtrs and corneal dysplasias; CDyps) have multifactorial etiologies and risk factors. For example, epithelial basement membrane dystrophy is thought to be degenerative rather than congenital; posterior polymorphous corneal dystrophy can present unilaterally; and Schnyder corneal dystrophy can have systemic effects in patients [3]. The International Classification of Corneal Dystrophies (IC3D), last updated in 2024, currently lists 26 distinct degenerative dystrophies and serves as a standard for distinguishing the various pathologies of the cornea based on anatomical involvement. Structurally, the cornea can be divided into five distinct layers. From the outermost to the innermost layer, they are the squamous epithelial layer, anterior basement (Bowman’s) membrane, collagen and keratocyte stromal layer, posterior basement membrane (Descemet’s), and endothelial layer. Pathogenesis is typically due to erosions or accumulation of foreign material within one or more of these five layers. Given these anatomically distinct layers in the cornea, the IC3D has four broad categories of CD: epithelial and subepithelial, epithelial–stromal, stromal, and endothelial. Previous work has elucidated many aspects of CDtrs, but the genetic contributions to this group of pathologies are incompletely understood. For example, even though many forms of CD have been associated with the Transforming Growth Factor Beta (TGF-ß)–Induced gene located on 5q31.1, the biological explanation of how one gene can have multiple phenotypic presentations in a single tissue structure is unclear. As such, the IC3D also describes categorizations based on the degree of our current genetic understanding of each specific dystrophy. Category 1 includes dystrophies with documented individual gene mutations. Category 2 describes dystrophies that have been mapped but without documented individual gene mutations. Category 3 groups dystrophies without an identified chromosomal locus, and category 4 is used for poorly defined dystrophies. While most dystrophies described by the IC3D are classified in category 1, there are some classified within categories 2–4, and others are grouped under more than one category, depending on subtype. Thus, a knowledge gap exists regarding the genetic underpinnings of the various forms of CD, some of which may be single-gene disorders, while others may be multigenic and/or with environmental factors. This study aimed to identify candidate CD genes in humans by studying knockout mice with targeted deletions of orthologous genes that exhibited statistically significant corneal abnormalities. Further, these data serve as a fundamental step to elucidate previously unknown genetic etiologies and molecular pathways of CDyps and CDtrs.

In vivo analysis of knockout mice has proven to be a powerful tool to investigate the biological mechanisms of disease. Analyzing mouse genes has provided useful insights into genetic abnormalities in humans. The International Mouse Phenotyping Consortium (IMPC) is a worldwide organization of 21 centers that are producing, comprehensively phenotyping, and cryopreserving for distribution single-gene knockout mice for every human orthologous protein-coding gene in the mouse genome. Knockout mice are produced by CRISPR/Cas9 editing [4] or homologous recombination in mouse embryonic stem (ES) cells [5]. Males and females for each unique single gene knockout mouse line undergo a series of in vivo, biochemical, molecular, and pathological phenotyping analyses and are compared to age, sex, and genetic-background-matched wild-type (WT) control mice. All data are robustly quality-controlled and statistically analyzed with results uploaded to a publicly available online database (mousephenotype.org) [6, 7]. The work of the IMPC has led to a more complete understanding of genotype-phenotype relationships and may provide insight into the genetic cause, contribution, and mechanism of human diseases. In this study, we queried IMPC data (Data Release 20.1; published 12 December 2023) for all mouse lines with corneal abnormalities and other systemic co-phenotypes in each line. Each mouse line associated with a corneal abnormality was considered a candidate CD gene. We performed a literature search to determine the extent to which each gene was previously implicated in corneal pathology. We used bioinformatic tools to determine the known cellular and molecular functions of each of these genes and to predict interactions between them and established human CD genes. Our approach also screened for novel genes and pathways not previously implicated in corneal dystrophy and corneal dysplasia that may have relevance in human disease processes.

## Materials and methods

### Animals and phenotyping

The IMPC has previously published how single-gene knockout mice are produced and phenotyped [6, 8] at each production center. Production centers disrupt protein-coding genes in the mouse genome, perform genetic quality control on the mouse line, and then generate phenotype-ready cohorts of 7 female and 7 male mice of each mutant line for phenotyping in parallel with age- and sex-matched WT control mice produced at the same Center. The IMPC initially used a gene trap with lacZ reporter but have now switched to using CRISPR/Cas9 technology. Data from all knockout mice, both the gene trap and the Cas9 methods, have been included in this study. For Data Release 20.1 (published 12 December 2023; queried 17 April 2024), there were 8,707 phenotyped unique genes, 9,393 phenotyped mutant lines, and 104,530 phenotype hits (*p* < 0.0001). As of data release 12, the IMPC applies appropriate statistical methods for each data type via Fisher’s exact test for categorical data and a linear mixed model for continuous data. More information can be found at https://www.mousephenotype.org/help/data-analysis/statistical-analysis/. All procedures at each IMPC center adhered to local, state, and national regulatory guidelines, based on the standards of Animal Research: Reporting of In Vivo Experiments (ARRIVE) guidelines, a list of recommendations to standardize and improve the quality and reproducibility of animal research. A Housing and Husbandry protocol was also followed, which contains a collection of mandatory and optional procedures to be used during international mouse experimentation [8]. Guidelines can be accessed at https://www.mousephenotype.org/about-impc/animal-welfare/. All procedures on live animals were reviewed and approved by associated institutional animal care and use committees (IACUC), animal care committees (ACC), or equivalent. Ocular phenotyping takes place at week 15–16 postnatal. Images of knockout mice were obtained via the IMPC phenotyping center where the mice were generated and examined.

The IMPC produces mice using either Cas9- or ES cell-derived mouse lines in the C57BL/6 N strain background [9] Phenotypes are described using standardized mammalian phenotyping ontology terms developed by the Mouse Genome Informatics group (https://www.informatics.jax.org/*).* Zygosity is also assigned as homozygous (HOM), heterozygous (HET), or hemizygous (HEM). More information can be found on the IMPC website at http://www.mousephenotype.org.

Mice are euthanized by CO2 asphyxiation followed by exsanguination via cardiac puncture in accordance with the UC Davis-IACUC approved protocol and euthanasia guidelines consistent with recommendations of the American Veterinary Medical Association (AVMA) 2020 Edition of the Guidelines for the Euthanasia of Animals.

### Bioinformatics

We initially formed our list of genes by querying all available phenotypes recorded by the IMPC that included the word “cornea.” We then downloaded all lines for each cornea phenotype and compiled them into one list. After eliminating redundant genes, we arrived at a single master list which formed the basis of our final spreadsheet of cornea gene hits. Abnormal corneal phenotypes were confirmed by manually searching each gene in the IMPC’s online dataset for independent confirmation of corneal abnormalities, yielding 213 candidate CD genes. A review of the current literature was conducted for previously published mouse models of these genes and associated corneal phenotypic abnormalities in humans. A parallel literature search was performed to generate a comprehensive list of previously published known CD genes, which yielded 48 established CD genes. While several of these genes came from the IC3D’s documented list of CD genes [3], a few genes were identified more recently and included [2, 10–16]. Human orthologues of all mouse CD genes were also analyzed for predicted protein-protein interactions and pathway functions using the Search Tool for the Retrieval of Interacting Genes/Proteins (STRING), Protein Analysis Through Evolutionary Relationships (PANTHER), and Kyoto Encyclopedia of Genes and Genomes (KEGG), respectively [17–21]. STRING provides confidence scores to provide comparisons between different interactions of genes. We limited our analysis to interactions which had thresholds of ≥ 0.7. Two miRNAs (one candidate, mir-96, and one CD gene, mir-184) do not appear in the STRING analysis, as well as the candidate genes Ngp and Stras6l, for which no functional human ortholog was identified.

All knockout lines were carefully manually curated using data from the web portal to confirm the presence of corneal abnormalities. Some lines were labeled as corneal phenotypic hits but upon further inspection had absent data for the ocular morphology phenotyping test. Any such genes erroneously labeled as significant by the software have been excluded from this study.

### Histopathology

A complete necropsy was performed and abnormal findings were recorded and annotated using the standardized IMPC Gross Pathology ontology [9]. When possible, macro-images of gross abnormalities were captured. Tissue samples collected at necropsy were immediately immersed in fixative (usually 10% neutral buffered formalin) and prepared for histopathological examination by a veterinary pathologist. Parasagittal sections of eyes were sectioned at 5 μm thickness and stained with hematoxylin and eosin (H&E). These mice were maintained using the approved animal protocols from UC Davis. More information can be found at https://www.ucdavis.edu/news/animals-research-and-teaching-uc-davis.

## Results

From our query of the IMPC database, we recorded the phenotype(s), zygosity, and P-values of all corneal phenotypes associated with each of the 213 genes. All corneal dysmorphologies were detected in 16 week-old mice when ocular exams were performed in the standardized IMPC phenotyping pipeline. This set of 213 genes included 14 genes previously associated with mouse CDs and 16 genes previously associated with human CDs, and 7 genes associated with both. These 16 genes were noted to be associated with human CDs and did not necessarily cause their associated abnormalities. For the established human CD genes, 45 out of these 48 genes were not found in the candidate CD gene list, either due to no knockout mouse lines having been generated (24 genes) or due to no significant corneal phenotype recorded by the IMPC (21 genes). Three established human CD genes (*FOXE3*, *PAX6*, *PITX2*) were found in the candidate CD gene lists.

**Fig. 1:**
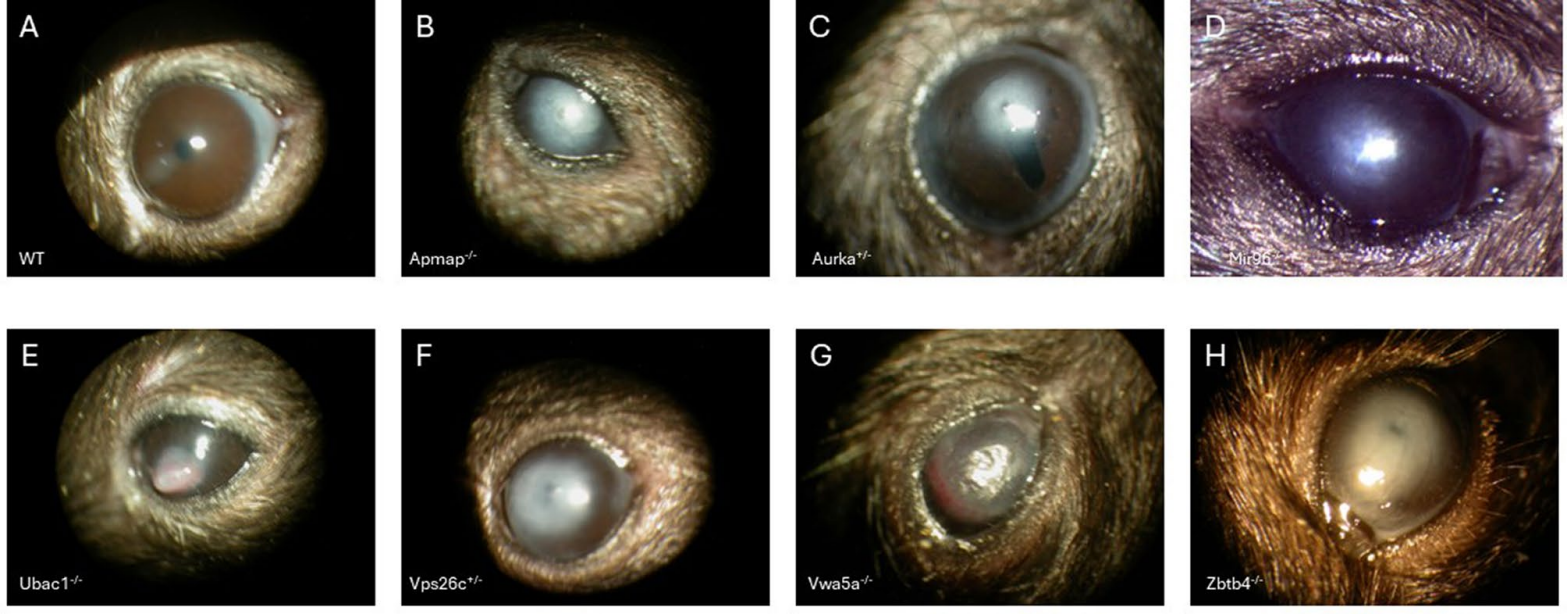
Example phenotypes of various knockout lines with corneal abnormalities documented by external color photography. Top Row: WT, Apmap^−/−^, Aurka^+/−^, Mir96^−/−^ Bottom Row: Ubac1-/-, Vps26c+/-, Vwa5a-/-, Zbtb4-/-

The 213 knockout mouse lines in the IMPC dataset associated with clinical corneal pathology were annotated with the one or more of the following ontology terms: sclerocornea, fused cornea and lens, increased cornea thickness, decreased cornea thickness, corneal vascularization, corneal opacity, corneal deposits, cornea ulcer, and/or abnormal cornea morphology not otherwise specified. Many genes demonstrated sexual dimorphism, with phenotypes achieving statistical significance in only one sex. A full list of candidate CD genes and details regarding specific abnormalities are provided in Supplemental Table 1. Only one zygosity was examined for all genes, with 136 HOM genes, 75 HET genes, and 2 HEM genes. Examples of corneal abnormalities of select mouse lines documented by image capture are shown in Fig. 1. The 48 established human clinical CD genes are compiled in Supplemental Table 2.

Some mouse lines demonstrated sexually dimorphic CDs, achieving statistical significance only in one sex. Of these, 60 lines showed statistically significant corneal abnormalities only in males while 84 lines showed statistically significant corneal abnormalities only in females. There were 3 genes with both male and female dimorphism (*Aspa*, *Barx2*, *Pfn1*). Interestingly, in these 3 lines, the specific corneal abnormality was different between sexes. For example, *Barx2* knockout mice had corneal vascularization in males but abnormal cornea morphology in females. Further study is required to confirm the degree of sexual dimorphism and its basis in each case. Details of each line are shown in Supplemental Table 1.

**Fig. 2:**
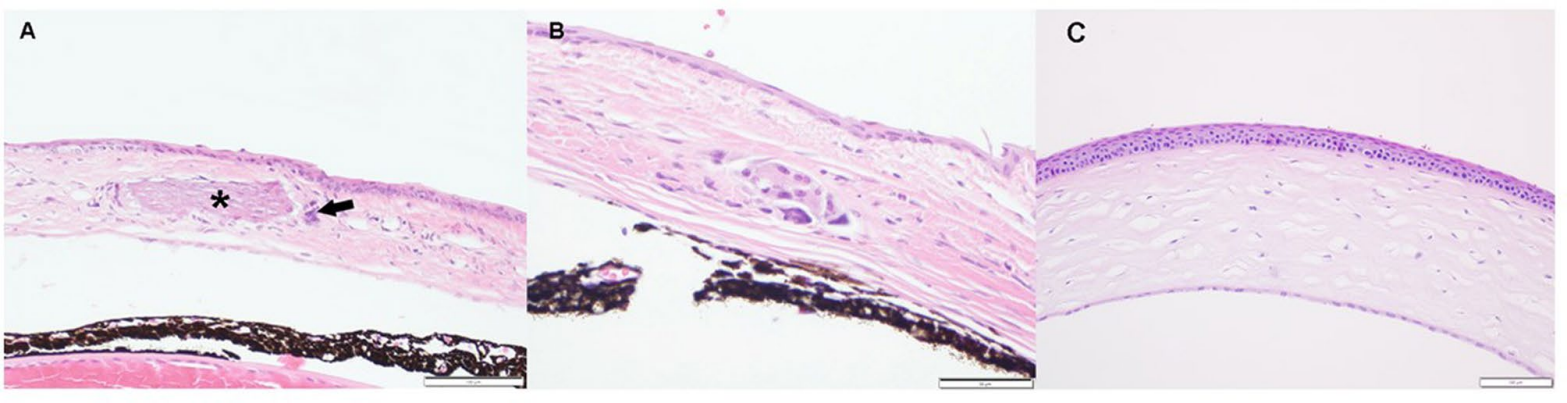
The corneal plaques in Nfil3-deficient mice (**A**, magnified view in **B**) represented foci of mid-stromal mineralization (asterisk) that elicit a variable degree of granulomatous response (arrow). Control corneal tissue from WT mice is shown in panel **C**

**Fig. 3:**
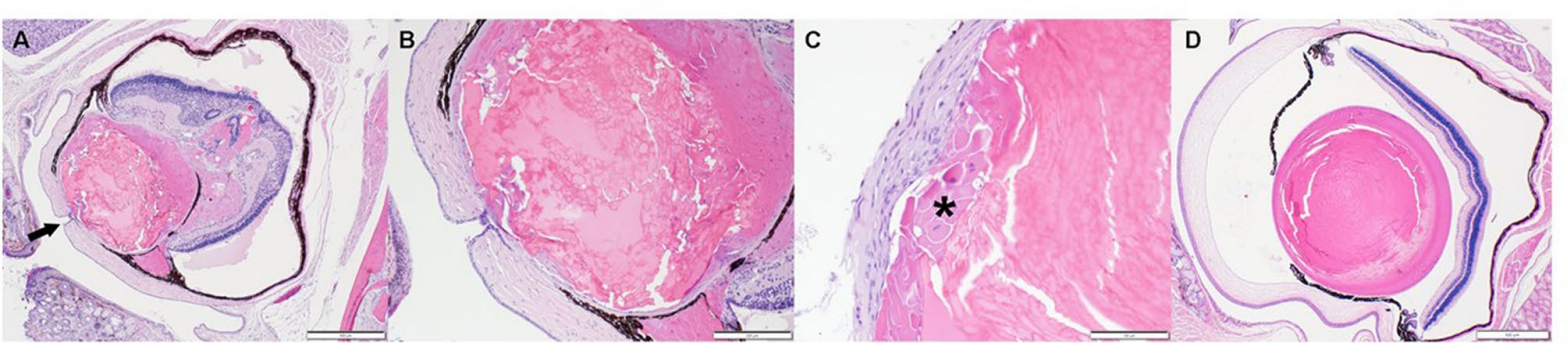
Pax6-deficiency manifests in the eyes as (**A**, magnified view in **B**) microphthalmia with a central corneal defect (black arrow in **A**), subcapsular cataract (asterisk in **C**), persistent hyaloid vasculature, anterior and posterior synechiae, retinal detachment and pthisis bulbi. The corneal defect (**B**) is covered by corneal epithelial downgrowth

We were also able to identify histopathological examples of corneal abnormalities from two candidate genes (*Nfil3*, *Pax6*). *Nfil3* knockout mice had corneal plaques due to mid-stromal foci of mineralization (Fig. 2). Deletion of *Pax6* resulted in embryonic lethality and anophthalmia in homozygous (HOM) embryos, and 16-week-old *Pax6* heterozygous (HET) animals had multiple congenital defects including a central corneal defect with persistent lenticular involvement (Fig. 3).

After identifying the set of candidate (mouse) and established (human) CD genes, we analyzed the human orthologues of the mouse candidate CD genes and the established human CD genes separately using STRING to determine potential protein-protein interactions within the genes of each set. Two candidate CD genes (*NGP*,* STRA6L*) and one established CD gene (MIR-184) were not in the STRING database and therefore could not be included in the analysis. Five candidate genes were not found in STRING so alternates were used in their place (GPR115 -> ADGRF4, AHSA2P -> AHSA1, IFI27L2A -> IFI27L2, SKIC2 -> SKIV2L, ZFP395 -> ZNF395).). All queried and unqueried terms are listed in Supplemental Table 3.

**Fig. 4:**
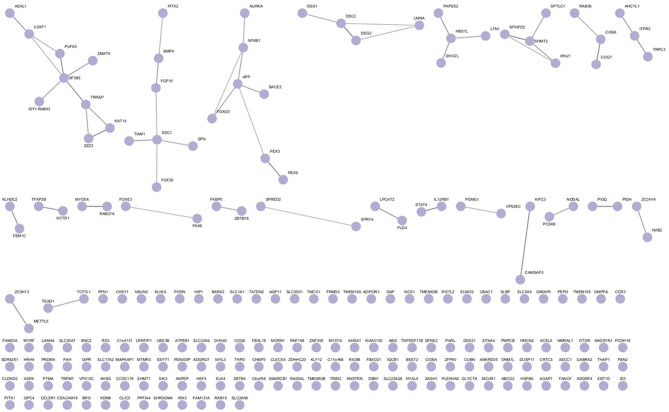
STRING analysis of protein-protein interactions between 210 out of the 213 candidate genes, with Organism set to Homo sapiens, and using the settings Network Type = full STRING network, Required score = high confidence (0.7) and FDR stringency = medium (5%). Two proteins (NGP, STRA6L) and one micro-RNA (MIR96) were omitted from this analysis since they were not included in the STRING tool

**Fig. 5:**
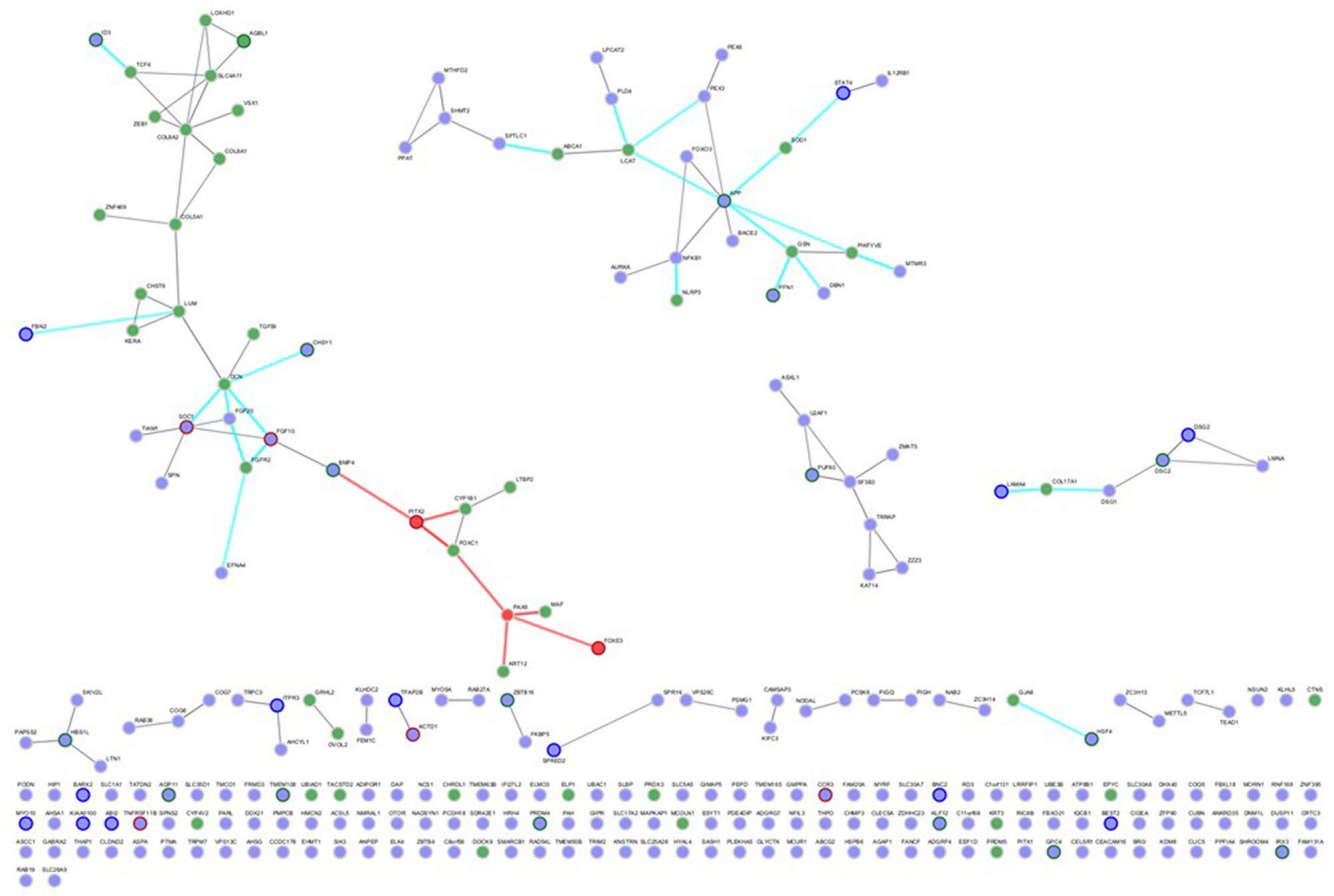
STRING analysis of protein-protein interactions between 210 Candidate genes (Purple) and 46 of the 48 gold standard genes (Green) (CNA1 was not present in STRING, nor the micro RNA miR-184), with Organism set to Homo sapiens, and using the settings Network Type = full STRING network, Required score = high confidence (0.7) and FDR stringency = medium (5%). PAX6, PITX2 and FOXE3, the three genes present in both lists, are represented in red. Nodes corresponding to genes for which there were PubMed reports of phenotypes in human, mouse or both are circled respectively in dark green, blue or dark red. Edges are color-coded in red when one node corresponds to one of the three common genes and in cyan when linking the two gene sets

The resulting interactome from STRING for the candidate CD genes is shown in Fig. 4. We found several clusters of predicted interactions between proteins within this group. We then determined predicted interactions between human orthologues of mouse candidate CD genes and established human CD genes by merging the datasets and analyzing the combined gene list in STRING. As can be seen in Fig. 5, numerous candidate genes (purple) have predicted interactions with established CD genes (green), with three genes (*PAX6*,* PITX2*,* FOXE3*) found in both lists (red). This results in a dense network with intermingled connections, 110 nodes and 106 edges, between candidate and established CD genes. In accordance with STRING’s confidence scores, 66% are high confidence (0.7 ≤ score < 0.9) and the remaining 34% are highest confidence (score ≥ 0.9). As MIR184 (IC3D) and MIR96 (candidate) were excluded for being pseudogenes, we looked to downstream targets that could be included in the STRING analysis, combining a search with Perplexity AI and PubMed (see supplemental information). This led to the inclusion of 25 genes for MIR184 (AKT1S1, AKT2, BCL2, CARM1, CDC25A, CRTC1, CTNNB1, DLX1, FOXO3, FZD7, IGF1R, INPPL1, ITGB4, JUN, LASP1, MYC, NFATC2, NKX6-1, NUMBL, SLC7A5, SND1, STC2, TNFAIP2, TP63, TSC2) and 31 genes for MIR96 (AKT1, AKT2, AKT3, AQP5, CELSR2, EHD1, FOS, FOXO1, FRS2, GFI1, IKZF2, KCNA10, MYO3A, MYRIP, NR3C1, OCM, ODF2, PAK1, PIK3R1, PRKCE, PTEN, PTPN9, PTQRQ, RAB2a, RARG, RYK, SEMA3E, SLC26A5, SLC52A3, SNAP23, ZEB1) which did extend the size of the larger connected cluster, but had only a limited impact on the number of isolated genes: 12 of the established genes in both cases, 124 vs. 131 of the candidate genes with or without the inclusion of micro RNAs targets, respectively (Supplementary Fig. 1).

From our literature search, we found that out of these 213 candidate genes, 14 (7%) were previously implicated in mouse corneal abnormalities, 16 (8%) were previously implicated in human corneal disorders, and 7 genes implicated in both for a total of 37 (17%) known genes. This left 176 of 213 (83%) genes not previously implicated in mouse or human corneal disease. Reference Pubmed IDs for previous studies are provided in Supplemental Table 1.

To determine the expression of candidate CD genes in the human cornea, we used publicly available online RNAseq data sets (https://plae.nei.nih.gov/) [22]. Each gene was queried on the PLatform for Analysis of scEiad (plae) ocular meta-atlas. Of the 213 orthologs of candidate mouse CD genes, 131 (62%) were expressed in human cornea, consistent with potential human corneal disease relevance. Furthermore, of the 37 genes previously implicated in corneal pathology, 25 (68%) were expressed in human cornea. Corneal tissue expression is annotated in Supplemental Table 1.

**Fig. 6:**
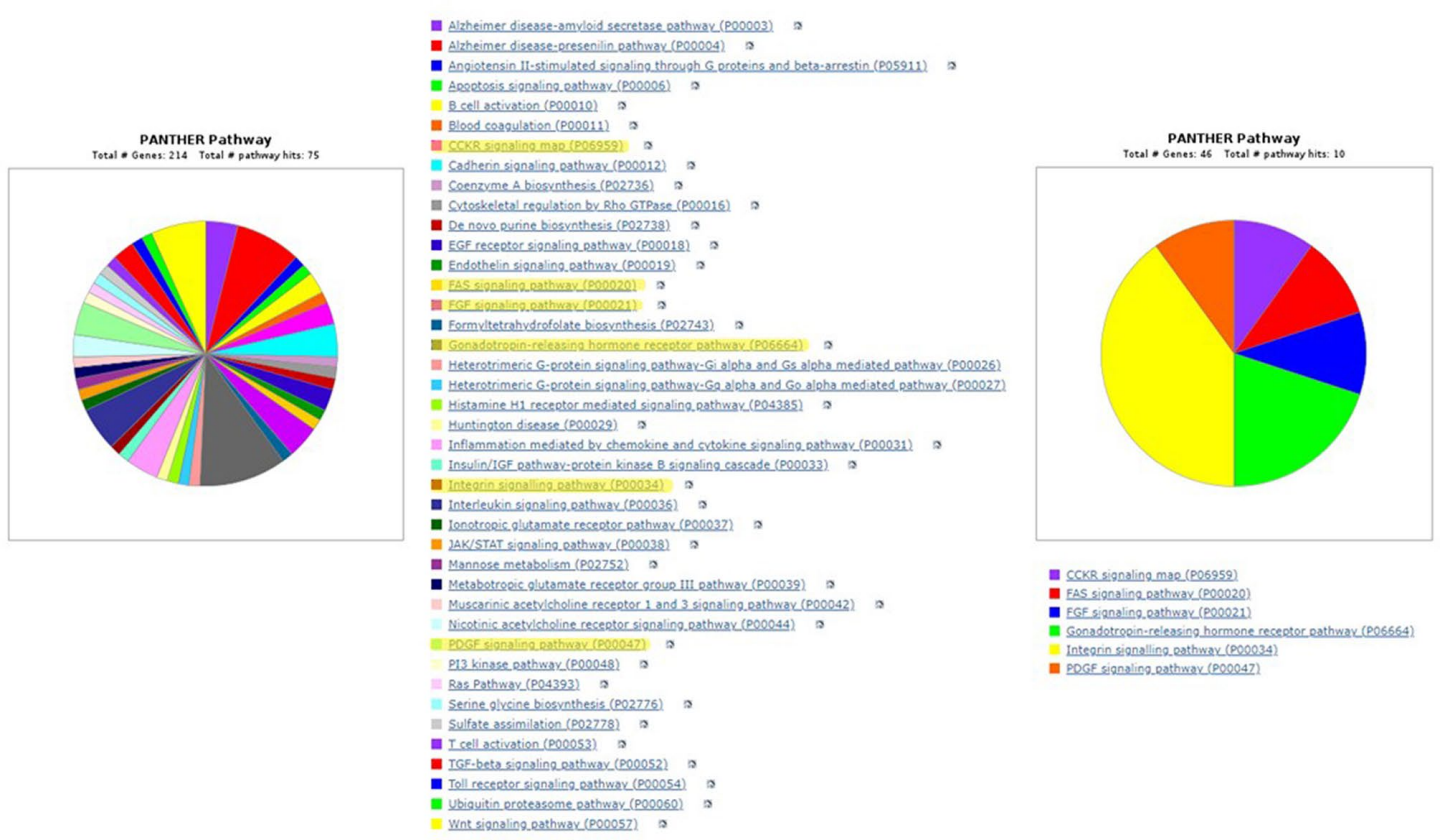
Molecular pathways of 213 candidate genes and 48 gold standard genes using the PANTHER tool. All gold standard CD gene pathways were found within the candidate pathways chart (highlighted)

Next, we used PANTHER to annotate molecular pathways in which candidate CD genes are involved and compared them to a similar analysis of the established CD genes. The PANTHER query was not able to give information regarding two established CD genes (CNA1, *MIR-184*). Our search resulted in associated signaling pathways for the candidate CD genes (Fig. 6). Established CD genes have been implicated in pathways for CCKR signaling, FAS signaling, FGF signaling, gonadotropin-releasing hormone receptors, integrin signaling, and PDGF signaling. PANTHER analysis also identified candidate CD genes in each of these pathways as well as other pathways not previously implicated in CD shown in Fig. 6.

**Fig. 7:**
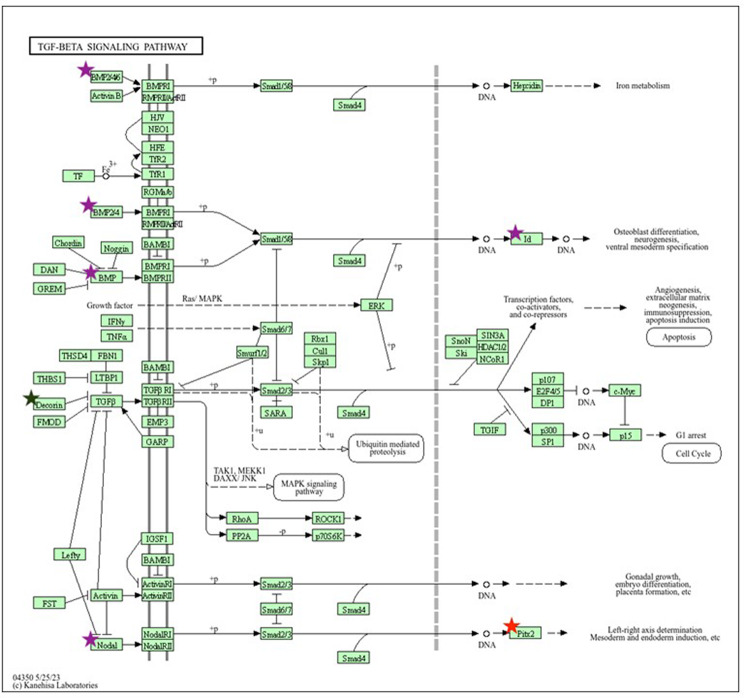
KEGG pathway for TGF-Beta signaling. Stars indicate genes from either the candidate CD list (purple), established CD gene list (green), or both (red)

We then explored specific gene and signaling pathways. Using KEGG, we conducted a search through cell pathway maps with documented functions and signaling cascades to link candidate CD genes to cellular functions. Due to limitations of the KEGG database, we were unable to query two candidate CD genes (*NGP*,* STRA6L*) and one established CD gene (*MIR-184*). We used KEGG to show relationships between candidate and established CD genes by merging them. Three candidate and two established CD genes appear to be involved in TGF-ß signaling (Fig. 7). Furthermore, five candidate genes and three established genes appear to be involved in regulation of actin cytoskeleton, one candidate gene and 4 established genes appear to be involved in protein digestion and absorption, three candidate genes and no established genes appear to be involved antifolate resistance, and 3 candidate genes and 1 established gene appear to be involved with peroxisomes (Supplemental Figs. 2–5).

## Discussion

In this study we identified 213 out of 8,707 unbiased single-gene knockout mouse lines produced and phenotyped by the IMPC that were reported to have corneal abnormalities, suggesting by extrapolation that ~ 1% of the mammalian genome is potentially relevant, directly or indirectly, to corneal pathology.

### Phenotypes

For the genes that displayed sexual dimorphism, there was a trend toward statistical significance in the other sex, but the P-value of < 0.0001 was not met. Sexual dimorphism is not unprecedented in corneal disease since Fuchs corneal endothelial dystrophy is less common in men than women [23]. Even within the same Fuchs pedigree, phenotypes can manifest significantly differently between the two sexes. Sexual dimorphism was seen in 146 of 213 lines (69%).

Out of the 24 established human CD genes which did have an IMPC knockout mouse line, 21 did not have an abnormal cornea phenotype. This may be attributed to the young age (~ 16 weeks) when mice underwent ocular phenotyping, the high-throughput nature of the ocular phenotyping employed by the IMPC, or to species differences. Nine of 21 established human CD genes were associated with lethality during the embryo stage or postnatal stage, which precluded ocular morphology assessment. Another possible reason for the lack of phenotype is knockout mice cannot model missense variants that may result in gain of function or dominant negative effects.

There were 75 mouse lines that had significant phenotypes in HET adult animals. These were associated with HOM embryonic lethality (24 of 75) or postnatal subviability (51 of 75) so no statistically significant clinical evaluation of the eye or other adult phenotyping was possible. These mutations likely follow a dominant inheritance pattern, leading to haploinsufficiency in the knockout mice.

### String

The candidate CD genes displayed several clusters of potential interactions. In total, 73 of the 213 queried candidate CD genes were connected to at least one other gene. When we merged the candidate gene set with the established human CD genes using STRING, the number of isolated genes decreased only moderately, from 140 to 132. The three genes that appeared in both lists (*FOXE3*, *PAX6*, *PITX2*) were involved in 31 connections, 10 with candidate genes and 21 with established genes. This interconnectedness between these two gene sets supports the notion that these candidate CD genes may be relevant to clinical CD in humans.

### Panther

As mentioned previously, all signaling pathways in the established CD genes’ PANTHER analysis were also included in the candidate CD gene pathway list. The complete overlap of established CD signaling pathways in the candidate CD signaling pathways confirms the strategy of this study to identify CD genes using screening of single-gene knockout mouse lines. The PANTHER analysis of the candidate CD gene list provides several general processes that may be involved in corneal development or function, such as cytokine reception, protein signaling, and cell-cell interaction. The PANTHER results, along with the STRING analysis, reveal potential mechanistic connections between candidate and established CD groups, suggesting shared cellular functions between genes in both groups. These shared cellular functions support the conclusion that the candidate CD gene list is involved in human corneal pathology.

### KEGG

When using KEGG to identify cellular pathways, we found involvement of both candidate and established CD genes in TGF-ß, actin cytoskeleton, antifolate resistance, peroxisomes, and protein digestion and absorption signaling. TGF-ß signaling is of particular importance as many forms of CD have been associated with this pathway [24, 25]. All three isoforms of TGF-ß are expressed in the human cornea and are involved in corneal development and fibrosis modulation [24]. Several studies have been conducted to target this pathway therapeutically [25]. The candidate genes (*BMP4*, *ID3*, *NODAL*,) and established genes (*PITX2*, *DCN*) involved in this pathway may serve as important targets for future studies, as their involvement in TGF-ß signaling suggests a strong link to CD.

### Limitations

The size of the mouse cornea makes it difficult to identify layer-specific corneal abnormalities during the IMPC’s high throughput in vivo phenotyping pipeline. Further studies in each mouse line are necessary to confirm and clarify the corneal cell type, layer affected, and molecular mechanism underlying the corneal pathology. Some genes may be involved in ocular surface biology (e.g. tear-film stability), corneal wound healing, ocular inflammation, or even behavioral abnormalities which may predispose to ocular trauma leading to acquired corneal phenotypes. Furthermore, data analysis and interpretation are limited to identifying genes associated with CD; additional experimental studies are needed to confirm whether these genes are also causative of CD.

Not all genetic mutations that produce corneal pathology in mice will do so in humans. Many cases of CD in humans involve missense mutations resulting in gain of function or dominant negative effects. Several hereditary corneal dystrophies are caused by missense mutations that result in misfolded proteins. Due to the nature of these mutations, they cannot be modeled through targeted gene deletions and could be better elucidated via ENU mutagenesis screening. Future studies are required to confirm that our candidate genes contribute to corneal abnormalities in humans including follow-up experiments using other molecular biology or immunohistochemical tools. Another limitation of this study includes the use of CRISPR-Cas9 generated mice, as there is potential for off-target effects. However, this risk is probably modest, as a whole genome analysis of 163 gRNAs revealed that only 4.9% of guides have off-target cutting activity, which is much lower than random genetic variation [26].

## Conclusions

This study identified 213 candidate genes from 8,707 IMPC knockout mouse lines for corneal dysmorphology, both corneal dystrophies and corneal dysplasia, 176 of which have not been previously associated with mouse or human CD. Using STRING and PANTHER, we compared our candidate CD genes with 48 established CD genes which revealed several interrelationships that require further investigation. Our bioinformatic analysis predicted potential mechanisms of pathogenesis relevant to CD, ocular surface disease, and/or corneal wound healing worthy of further study. We hope this manuscript serves as a starting point for the field to pursue more detailed work in animal models and in patient populations now that genetic testing is becoming more economical, though still limited in global application.

## Electronic supplementary material

Below is the link to the electronic supplementary material.

Supplementary Material 1

Supplementary Material 2: Supplemental Table 1: A list of all candidate CD genes. Red highlights the 7 HET genes that were not associated with lethality or subviability. A coding system for phenotype systems is included. Table 2: A list of all established CD genes along with their effects of embryo and lethality phenotypes as noted by the IMPC. Table 3: A list of both candidate and established CD genes along with the names used to query in STRING and KEGG.

Supplementary Material 3: Fig. 1: STRING analysis of protein-protein interactions between 210 Candidate genes (Purple), 46 of the 48 established genes (Green), with the three common genes PAX6, PITX2 and FOXE3 represented in red, and some identified targets of the established miR-184 (Cyan) and the candidate miR-96 (Beige). The parameters were Organism = Homo sapiens, Network Type = full STRING network, Required score = high confidence (0.7) and FDR stringency = medium (5%). AKT1 (Pink) is both a target of miR-184 and miR-96, and ZEB1 (dark green) is both an established gene and target of miR-96. The seven newly connected candidate genes ADIPOR1, AQP11, CRTC3, GPC4, MAPKAP1, NSUN2 and SMARCB1 are in dark purple.

Supplementary Material 4: KEGG pathway for regulation of actin cytoskeleton. Stars indicate genes from either the Candidate CD list (purple), established CD gene list (green), or both (red).

Supplementary Material 5: KEGG pathway for protein digestion and absorption. Stars indicate genes from either the Candidate CD list (purple), established CD gene list (green), or both (red).

Supplementary Material 6: KEGG pathway for antifolate resistance. Stars indicate genes from either the Candidate CD list (purple), established CD gene list (green), or both (red).

Supplementary Material 7: KEGG pathway for peroxisome biogenesis. Stars indicate genes from either the Candidate CD list (purple), established CD gene list (green), or both (red).

## Data Availability

The datasets generated and/or analyzed during the current study are available in the International Mouse Phenotyping Consortium repository (https://mousephenotype.org).

## Abbreviations

CDs: Corneal dysmorphologies
CDtrs: Corneal dystrophies
CDyps: Corneal dysplasias
STRING: Search Tool for the Retrieval of Interacting Genes/Proteins
PANTHER: Protein Analysis Through Evolutionary Relationships
KEGG: Kyoto Encyclopedia of Genes and Genomes
IMPC: International Mouse Phenotyping Consortium
IC3D: The International Classification of Corneal Dystrophies
