# Supplementary material for: Systematic ocular phenotyping of 8,707 knockout mouse lines identifies genes associated with abnormal corneal phenotypes
Source: BMC Genomics. 2025 Jan 20;26:48. doi: 10.1186/s12864-025-11222-8 (PMC11744888; doi:10.1186/s12864-025-11222-8)
Supplement: Supplementary file 1 — Supplementary Material 1 [file 12864_2025_11222_MOESM1_ESM.docx]

As MIR184 (IC3D) and MIR96 (candidate) were excluded for being pseudogenes, we looked to downstream targets that could be included in the STRING analysis, combining a search with Perplexity AI and PubMed.

To identify targets of miR-96 and miR-184, we queried [Perplexity AI](https://www.perplexity.ai/), as it provides results substantiated with bibliographic references. Using the pro mode, we asked which genes have their expression regulated by either micro RNAs in mammals. This pointed to INPPL1 [1–3], ITGB4 [1, 2], AKT2, NFAT1 (NFATC2 in STRING), NUMBL and TSC2 [1, 4], BCL2, CARM1, CDC25A, CRTC1, DLX1, FOXO3, FZD7, IGF1R, JUN, LASP1, MYC, , NKX6.1, PRAS40 (AKT1S1 in STRING), SLC7A5, SND1, TNFAIP2 [1], TP63 [3], CTNNB1 [5]

A similar query for miR-96 indicated MYRIP [6], AQP5, FOS, FOXO1, GFI1, IKZF2, KCNA10, OCM, MYO3A, NR3C1, PTPRQ, SEMA3E, SLC26A5, SLC52A3 and ZEB1 (which is also one of the IMPC candidate gene) [7], CELSR2, ODF2 and RYK [8]. A further search with PubMed indicated as additional targets RARG [9], AKT1, AKT2, AKT3, EHD1, PAK1, PIK3R1, PRKCE, RAB1A and SNAP23 [10], FRS2 [11], PTEN [12] and PTPN9 [13]. This list is not exhaustive, and may contain indirect targets of either miR-184 or miR-96.

This did extend the size of the larger connected cluster, but had only a limited impact on the number of isolated genes: 12 of the established genes in both cases, 124 vs. 131 of the candidate genes with or without the inclusion of micro RNAs targets, respectively (Supplementary Figure 1).

1. Fattahi M, Rezaee D, Fakhari F, Najafi S, Aghaei-Zarch SM, Beyranvand P, et al. microRNA-184 in the landscape of human malignancies: a review to roles and clinical significance. Cell Death Discov. 2023;9:1–15.

2. Hughes AE, Bradley DT, Campbell M, Lechner J, Dash DP, Simpson DA, et al. Mutation altering the miR-184 seed region causes familial keratoconus with cataract. Am J Hum Genet. 2011;89:628–33.

3. Li J, Pan C, Tang C, Tan W, Zhang W, Guan J. miR-184 targets TP63 to block idiopathic pulmonary fibrosis by inhibiting proliferation and epithelial-mesenchymal transition of airway epithelial cells. Lab Invest. 2021;101:142–54.

4. Phua YW, Nguyen A, Roden DL, Elsworth B, Deng N, Nikolic I, et al. MicroRNA profiling of the pubertal mouse mammary gland identifies miR-184 as a candidate breast tumour suppressor gene. Breast Cancer Research. 2015;17:83.

5. Turovsky L, Kheshaiboun G, Yassen G, Nagosa S, Boyango I, Amitai-Lange A, et al. miR-184 represses β-catenin and behaves as a skin tumor suppressor. Cell Death Dis. 2024;15:1–10.

6. Soldà G, Robusto M, Primignani P, Castorina P, Benzoni E, Cesarani A, et al. A novel mutation within the MIR96 gene causes non-syndromic inherited hearing loss in an Italian family by altering pre-miRNA processing. Human Molecular Genetics. 2012;21:577–85.

7. Lewis MA, Di Domenico F, Ingham NJ, Prosser HM, Steel KP. Hearing impairment due to Mir183/96/182 mutations suggests both loss-of-function and gain-of-function effects. Disease Models & Mechanisms. 2021;14:dmm047225.

8. Lewis MA, Quint E, Glazier AM, Fuchs H, De Angelis MH, Langford C, et al. An ENU-induced mutation of miR-96 associated with progressive hearing loss in mice. Nat Genet. 2009;41:614–8.

9. Long MD, Singh PK, Russell JR, Llimos G, Rosario S, Rizvi A, et al. The miR-96 and RARγ signaling axis governs androgen signaling and prostate cancer progression. Oncogene. 2019;38:421–44.

10. Zolfaghari N, Soheili Z-S, Samiei S, Latifi-Navid H, Hafezi-Moghadam A, Ahmadieh H, et al. microRNA-96 targets the INS/AKT/GLUT4 signaling axis: Association with and effect on diabetic retinopathy. Heliyon. 2023;9:e15539.

11. Yang X, Liu H, Zhang Q, Liu K, Yu D, Zhang Y, et al. MiR-96 promotes apoptosis of nucleus pulpous cells by targeting FRS2. Human Cell. 2020;33:1017–25.

12. Park SE, Kim W, Hong J-Y, Kang D, Park S, Suh J, et al. miR-96-5p targets PTEN to mediate sunitinib resistance in clear cell renal cell carcinoma. Sci Rep. 2022;12:3537.

13. Hong Y, Liang H, Uzair-ur-Rehman, Wang Y, Zhang W, Zhou Y, et al. miR-96 promotes cell proliferation, migration and invasion by targeting PTPN9 in breast cancer. Sci Rep. 2016;6:37421.
