# Supplementary figures and images for: Systematic ocular phenotyping of 8,707 knockout mouse lines identifies genes associated with abnormal corneal phenotypes
Source: BMC Genomics. 2025 Jan 20;26:48. doi: 10.1186/s12864-025-11222-8 (PMC11744888; doi:10.1186/s12864-025-11222-8)

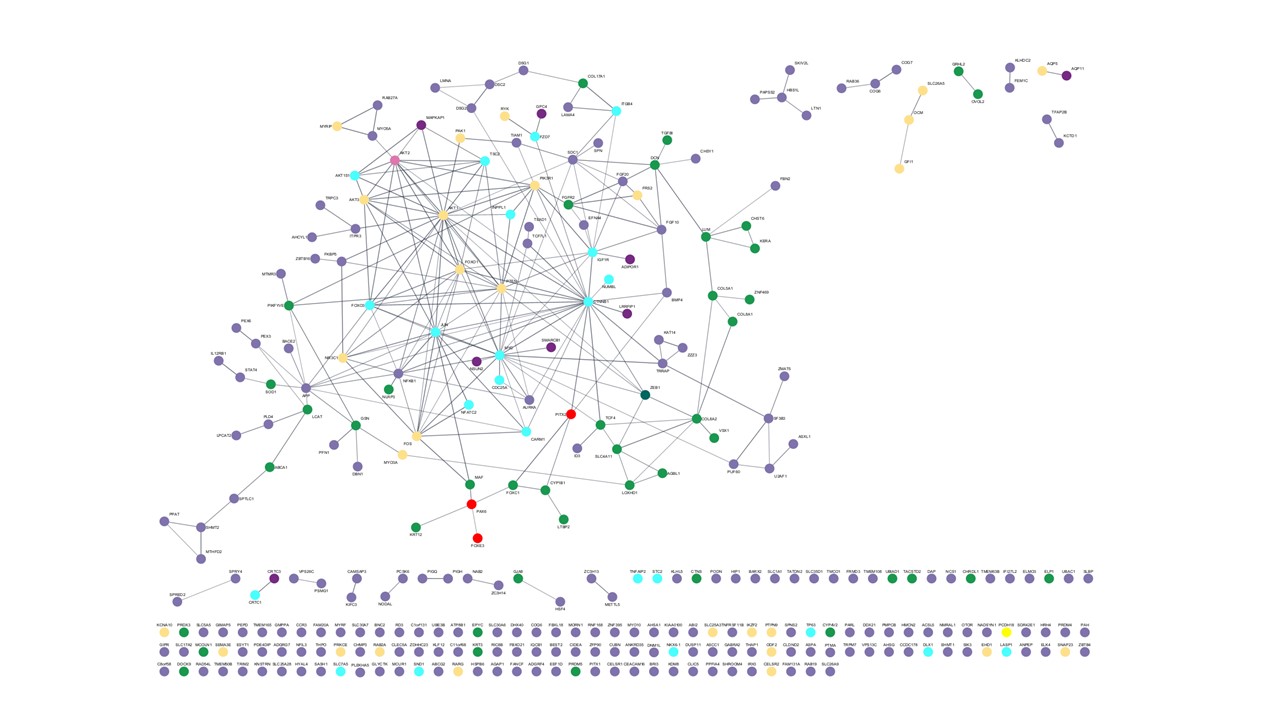

Supplement: Supplementary file 3 — Supplementary Material 3: Fig. 1: STRING analysis of protein-protein interactions between 210 Candidate genes (Purple), 46 of the 48 established genes (Green), with the three common genes PAX6, PITX2 and FOXE3 represented in red, and some identified targets of the established miR-184 (Cyan) and the candidate miR-96 (Beige). The parameters were Organism = Homo sapiens, Network Type = full STRING network, Required score = high confidence (0.7) and FDR stringency = medium (5%). AKT1 (Pink) is both a target of miR-184 and miR-96, and ZEB1 (dark green) is both an established gene and target of miR-96. The seven newly connected candidate genes ADIPOR1, AQP11, CRTC3, GPC4, MAPKAP1, NSUN2 and SMARCB1 are in dark purple. [file 12864_2025_11222_MOESM3_ESM.jpg]

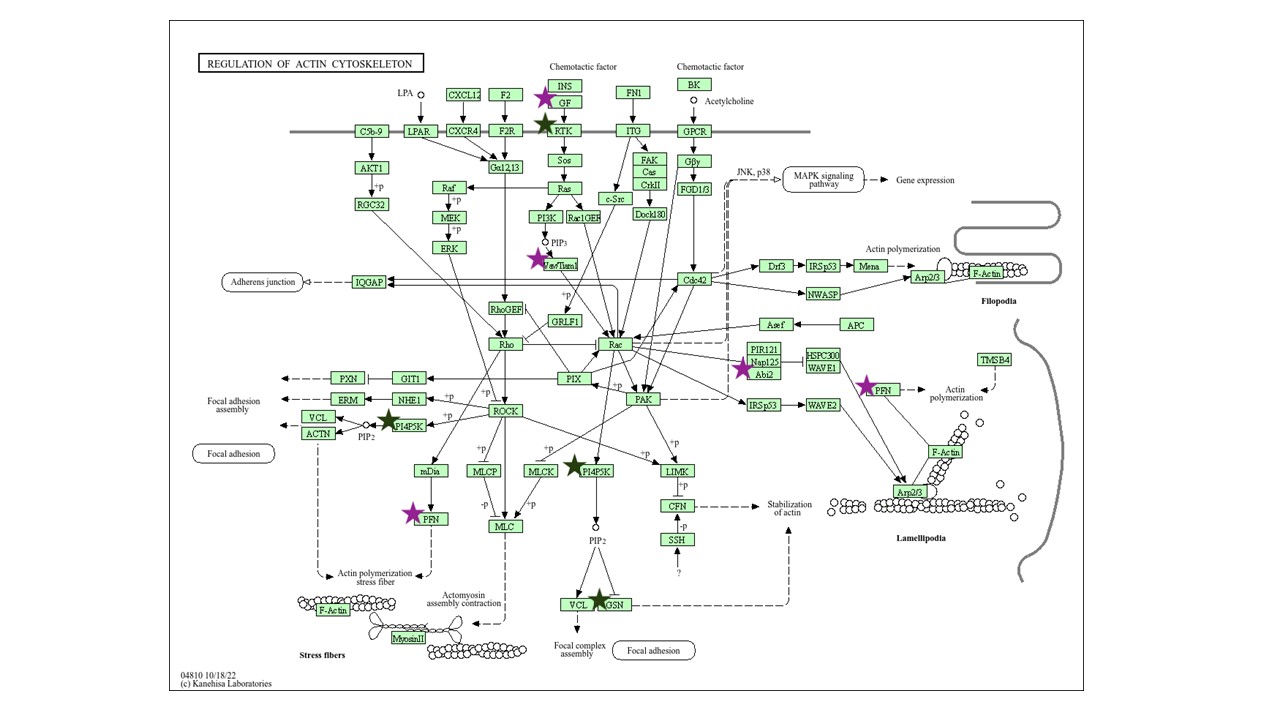

Supplement: Supplementary file 4 — Supplementary Material 4: KEGG pathway for regulation of actin cytoskeleton. Stars indicate genes from either the Candidate CD list (purple), established CD gene list (green), or both (red). [file 12864_2025_11222_MOESM4_ESM.jpg]

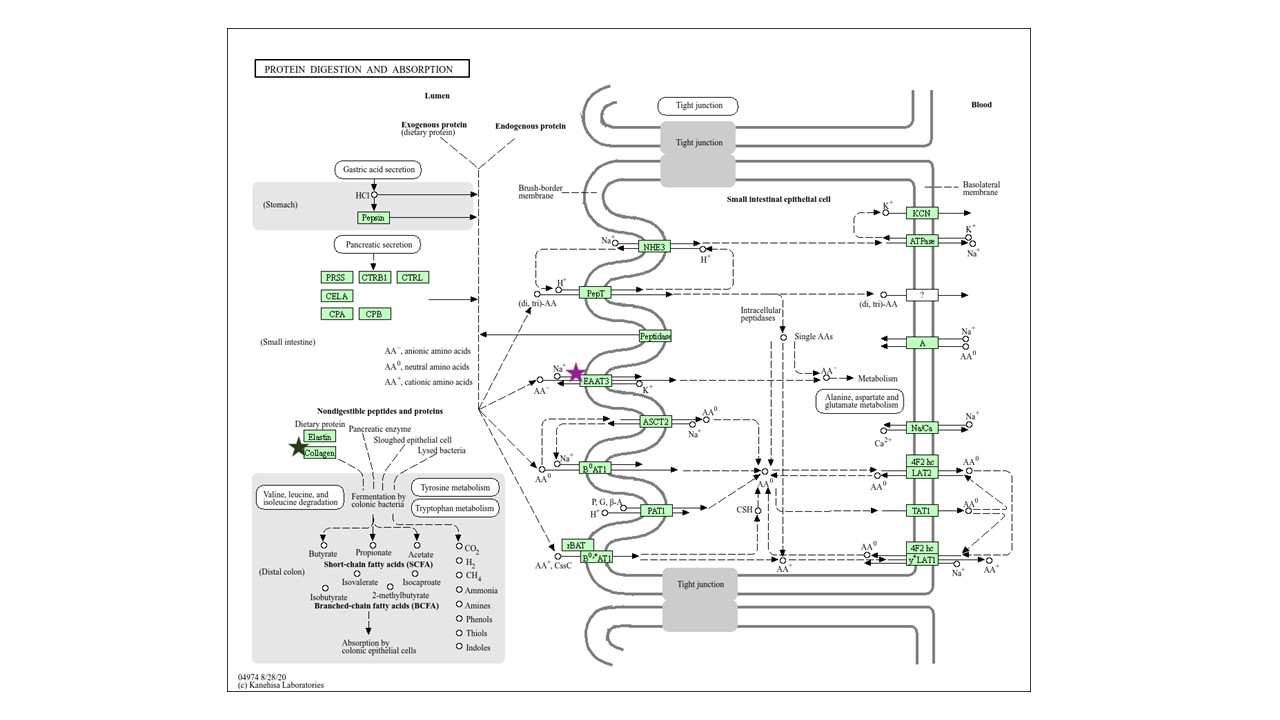

Supplement: Supplementary file 5 — Supplementary Material 5: KEGG pathway for protein digestion and absorption. Stars indicate genes from either the Candidate CD list (purple), established CD gene list (green), or both (red). [file 12864_2025_11222_MOESM5_ESM.jpg]

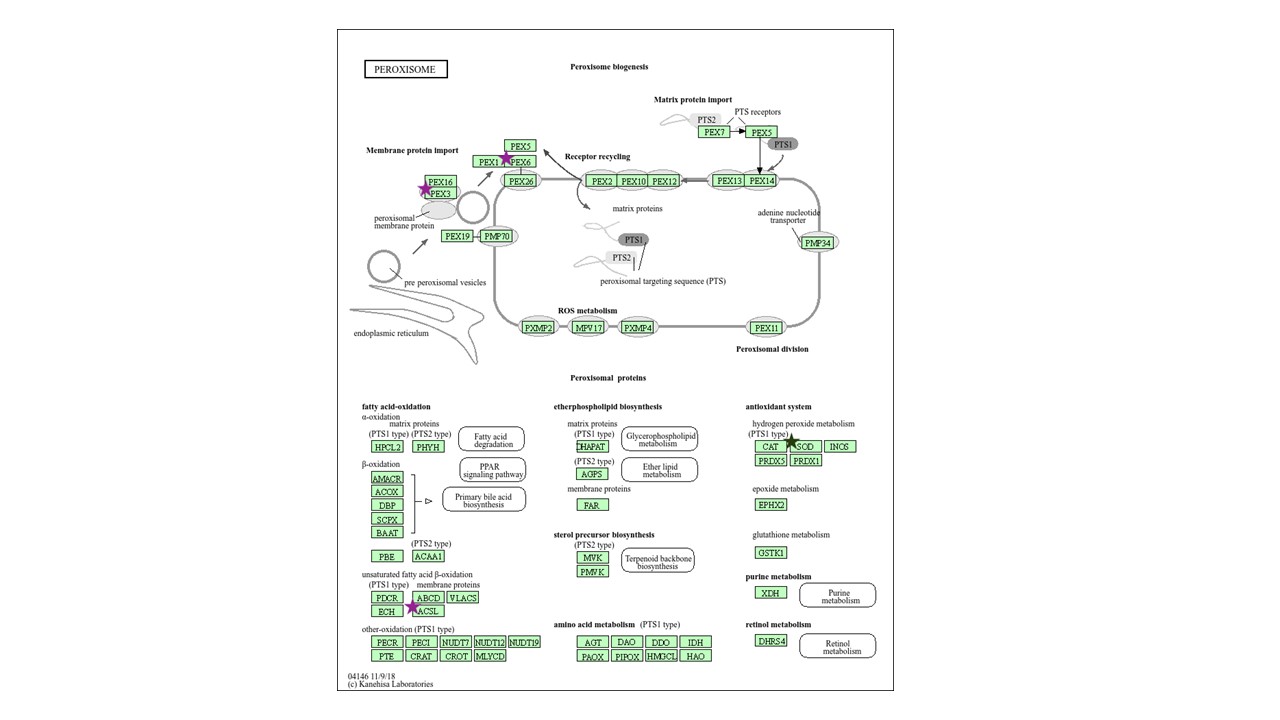

Supplement: Supplementary file 6 — Supplementary Material 6: KEGG pathway for antifolate resistance. Stars indicate genes from either the Candidate CD list (purple), established CD gene list (green), or both (red). [file 12864_2025_11222_MOESM6_ESM.jpg]

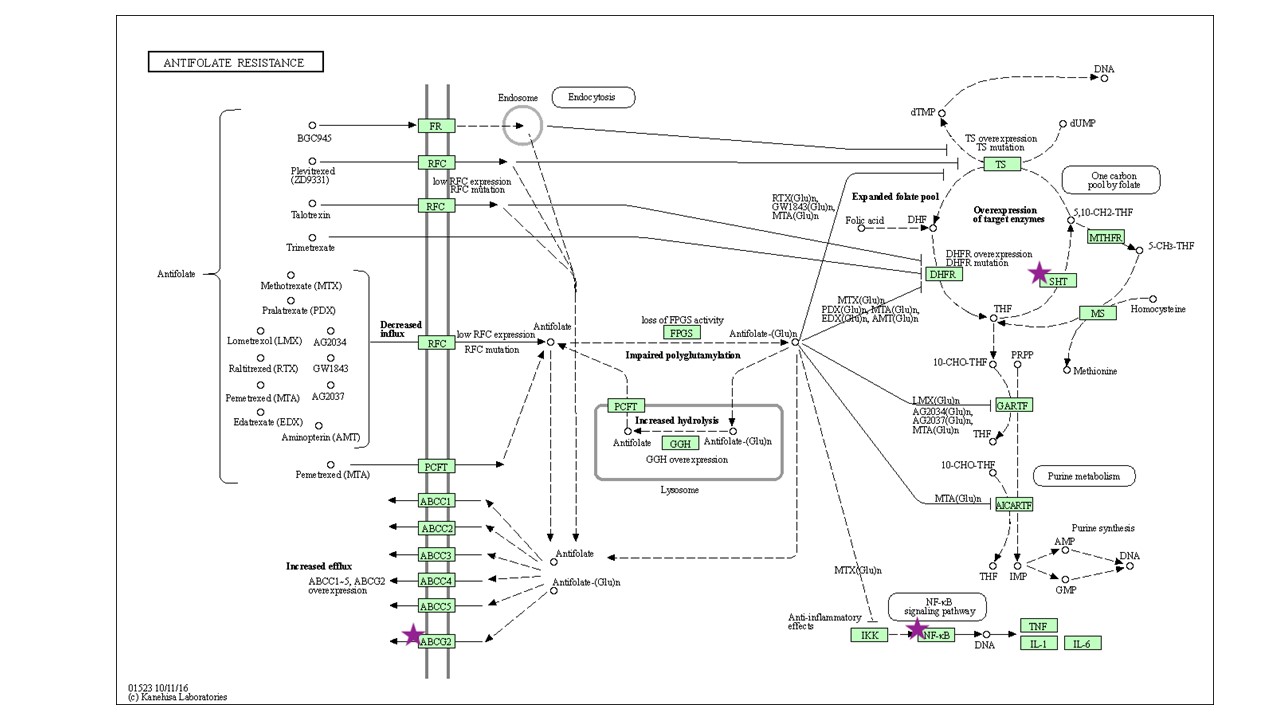

Supplement: Supplementary file 7 — Supplementary Material 7: KEGG pathway for peroxisome biogenesis. Stars indicate genes from either the Candidate CD list (purple), established CD gene list (green), or both (red). [file 12864_2025_11222_MOESM7_ESM.jpg]
